# Delusional disorder and its differentiation from schizophrenia: A narrative review

**DOI:** 10.1017/S0033291726103663

**Published:** 2026-05-15

**Authors:** Daniele Rossi Grauenfels, Mads Gram Henriksen, Julie Nordgaard

**Affiliations:** 1 AUSL di Bologna: Azienda Unità Sanitaria Locale di Bologna, Italy; 2 Central and Western Zealand Hospital, Denmark; 3 Center for Subjectivity Research, University of Copenhagen, Denmark; 4 Department of Clinical Medicine, University of Copenhagen, Denmark

**Keywords:** bizarre delusions, differential diagnosis, first-rank symptoms, paranoia, systematization

## Abstract

The nosological status and psychopathology of delusional disorder have been a subject of debate since Kraepelin distinguished it from schizophrenia and affective psychoses. Contemporary diagnostic manuals define delusional disorder primarily by the presence of delusions, offering limited guidance for its differentiation from schizophrenia. Notably, DSM-5 introduced a major, seemingly unexplained change by allowing bizarre delusions in delusional disorder, contrary to ICD-11, prior DSM editions, and classical descriptions. This narrative review revisits the seminal works of six classical authors (Kraepelin, Jaspers, Kretschmer, Sérieux and Capgras, and De Clérambault), who shaped the concept of delusional disorder (paranoia), and their detailed clinical cases of the disorder. All considered delusional disorder to be an independent psychotic disorder, characterized by chronic, systematized, nonbizarre delusions, preservation of personality, and minimal hallucinations, with a largely intact experiential framework outside of the delusional theme. Additional features such as delusional misinterpretations, illusions, and false memories were also emphasized in the classical literature. We examined these authors’ clinical cases of delusional disorder for the presence of delusional features characteristic of schizophrenia (delusional mood, first-rank symptoms, autistic-solipsistic delusions, and double bookkeeping), which index alterations in the structure of experience rather than mere delusional content. Such delusions were rarely found in the classical clinical cases of delusional disorder. Our findings highlight psychopathological distinctions between delusional disorder and schizophrenia, suggesting that schizophrenia involves a qualitative alteration of the experiential framework that is absent in delusional disorder. These findings raise concerns about the validity of the DSM-5 change, allowing bizarre delusions in delusional disorder.

## Introduction

Prior to Kraepelin’s seminal nosological work, the term ‘paranoia’ was used inconsistently to describe a broad spectrum of mental disorders (Lacan, 1975; Lévy, 2019). With Kraepelin’s diagnostic categories established in 1899, paranoia came to designate a mental disorder distinct from other psychotic disorders such as schizophrenia and affective psychosis (Munro, 1999; Kendler, 2018). Throughout much of the 20th century, ambiguity surrounded both the very name of the disorder, which, following Winokur (1977), eventually was renamed delusional disorder in DSM-III-R (American Psychiatric Association, 1987), and its nosological status as an independent psychotic disorder as championed by Kraepelin (1899, 1903, 1915). Some scholars, like Schneider, argued that what now is known as delusional disorder was in fact a subtype of schizophrenia (Schneider, 1959), while others, like Specht, argued that it was a form of affective psychosis (Specht, 1901). In the early 1980s, the nosological status of delusional disorder as an independent psychotic disorder gained renewed support, following influential reviews by Kendler (1980, 1982), and it has maintained this status in ICD-11 (World Health Organization, 2024) and DSM-5-TR (American Psychiatric Association, 2022).

Still, the differential diagnosis between delusional disorder and schizophrenia can be difficult (Kraepelin, 1903). In ICD-11 and DSM-5-TR, delusional disorder is defined by one positive symptom (delusion); all other diagnostic criteria are exclusionary. Delusions, however, are also characteristic of schizophrenia and other psychotic disorders. Crucially, the diagnostic manuals differ on a key issue, namely, regarding the type of delusion allowed in delusional disorder. While ICD-11 excludes delusions, traditionally considered characteristic of schizophrenia (‘experiences of influence, passivity or control’) (World Health Organization, 2024) (pp. 163, 185), DSM-5-TR does not restrict the type of delusion allowed in delusional disorder. This marks not only a difference from ICD-11 but also a major and, to our knowledge, unaddressed change in the conceptualization of delusional disorder from prior versions of DSM, which, since DSM-III, only has allowed nonbizarre delusions to occur in delusional disorder (American Psychiatric Association, 1980, p. 196; American Psychiatric Association, 1987, p. 202; American Psychiatric Association, 1994, p. 301; American Psychiatric Association, 2000, p. 329). Furthermore, DSM-5-TR even operates with a specifier for delusions with ‘bizarre content’ in delusional disorder. DSM-5 highlights this change but provides no reason for it (American Psychiatric Association, 2013, p. 810). Looking at the literature, we have not been able to identify studies warranting this change, neither in the period preceding the DSM-5 nor in the years following its publication in 2013.

The addition of the specifier for bizarre delusion in delusional disorder occurred alongside other changes in the section on Schizophrenia Spectrum and Other Psychotic Disorders in DSM-5, most notable in this context is the de-emphasis of the diagnostic significance of bizarre delusion for schizophrenia (American Psychiatric Association, 2013) as described by Heckers et al. (2013) and Tandon et al. (2013). However, it does not logically follow from the removal of bizarre delusions as *specific* to schizophrenia that bizarre delusions should be selectively permitted in delusional disorder, which in prior versions of DSM was defined by the presence of nonbizarre delusions only. Moreover, permission of bizarre delusion in other psychotic disorders such as bipolar disorder or major depressive disorder with psychotic features was not articulated in DSM-5, leaving the conceptual or empirical reasons for permitting bizarre delusion in delusional disorder unexplained.

In DSM-IV (p. 275) and DSM-5 (p. 87), delusions are considered ‘bizarre’ if they are clearly implausible, nonunderstandable, and not derived from ordinary life experiences. As emphasized by Cermolacce et al. (2010), this definition of bizarre delusion focuses mostly on the *content* of the delusion, following the trail of DSM-III, which stated that these delusions’ ‘content is patently absurd and has *no* possible basis in facts’ (p. 188). From DSM-III to DSM-5-TR, bizarre delusions thus overlap with experiences of influence, passivity, or control (e.g. thought insertion, withdrawal, or broadcasting, or delusions of control) (American Psychiatric Association, 2013, p. 87). Within the DSM framework, ‘nonbizarre’ delusions, by contrast, have a readily understandable content, e.g. delusions of being under police surveillance or suffering from an illness (American Psychiatric Association, 2013, p. 87).

Seeking to better differentiate, several studies have tried to disentangle the two disorders more clearly. Compared with schizophrenia, delusional disorder has been associated with fewer hallucinations (Hui et al., 2015), less severe negative symptoms (Marneros et al., 2012; Muñoz-Negro et al., 2017; Yassa & Suranyi-Cadotte, 1993), and delusions have been found to be characterized by a greater degree of conviction and less disorganization than in schizophrenia (Peralta & Cuesta, 2016). Other work (Hui et al., 2023) has proposed that age matching in delusional disorder and schizophrenia samples may account for some differences, leaving negative symptoms and impaired functional outcome as the most distinct markers of schizophrenia. Longitudinal studies further indicate that a proportion of patients initially diagnosed with delusional disorder are later rediagnosed with schizophrenia (Heslin et al., 2015). In parallel with these empirical findings, an important contemporary contribution is Kendler’s comprehensive review of textbook descriptions of paranoia and delusional disorder (Kendler, 2017), which documents substantial historical agreement on several defining features of delusional disorder, especially the emphasis on nonbizarre delusions and preserved functioning. Taken together, this body of work has considerably advanced descriptive knowledge of delusional disorder but offers limited guidance for differential diagnostic assessment, particularly with respect to the experiential structure in which delusions arise.

This study is complementary to Kendler’s contribution but differs in both its sources and analytical focus. We revisit the seminal writings of some of the authors, who originally shaped the concept of delusional disorder (paranoia), and we systematically analyze their detailed clinical cases. This approach allows us to examine not only how delusions were presented at a descriptive level, but also the experiential framework in which they were embedded, and to assess whether delusional disorder was understood to involve alterations of the structure and context of experience comparable to those characteristics of schizophrenia.

While the psychopathology of schizophrenia has been extensively researched for decades, the psychopathology of delusional disorder remains comparatively underexplored in contemporary research. The longstanding and narrow focus on delusion in the diagnostic criteria for delusional disorder has contributed to a situation where broader knowledge of delusional disorder has gradually slipped out of awareness. To reinstall such knowledge and improve differential diagnostic resources, we revisit key classical contributions of delusional disorder. Beyond rediscovering forgotten psychopathological features of delusional disorder, this approach allows us to assess whether or not delusional disorder was originally understood as involving alterations of the experiential framework as in schizophrenia. This approach further enables an assessment of whether the DSM-5 change, permitting bizarre delusions in delusional disorder, represents continuity with, or a departure from, the original conceptualization of this disorder.

## Aim

The aim of our study is to provide a psychopathological characterization of delusional disorder and its differentiation from schizophrenia based on theoretical considerations and clinical cases from the classical scholars, who shaped the concept of delusional disorder (paranoia).

## Methods

In classical psychiatric literature, six authors are widely considered to have made the greatest contributions to delusional disorder, providing either a general conceptualization of the disorder or of a specific subtype of it, and providing clinical cases. The authors are: *Emil Kraepelin* (‘Psychiatrie’, from the 6th edition of 1899 to the 8th edition of 1915; ‘Über paranoide Erkrankungen’, 1912; ‘Krankenvorstellungen: Paranoide Erkrankungen und Dementia praecox’, 1919; ‘Lectures on clinical psychiatry’, 1906); *Karl Jaspers* (‘Eifersuchtswahn’, 1910 [2015]); *Ernst Kretschmer* (‘Der sensitive Beziehungswahn’, 1918 [2016]); *Paul Sérieux and Joseph Capgras* (‘Les folies raisonnantes’, 1909); *Gaëtan Gatian De Clérambault* (‘Les psychoses passionelles’, 1942). We did not include works that merely discussed a specific aspect of the disorder, e.g. demonstration of a psychogenic cause for paranoia as in Lacan’s ‘De la psychose paranoïaque dans ses rapports avec la Personnalité’ (1975) or the independence of paranoia from affective disorders and other issues in Bleuler’s ‘Affectivity, Suggestibility, Paranoia’ (1912).

All these authors considered delusional disorder as a disorder independent from schizophrenia and affective psychosis. We read and analyzed their main works in which they deal with delusional disorder (paranoia) (section ‘Psychopathology of delusional disorder’). We also carefully read their clinical cases of delusional disorder (paranoia). Since De Clérambault makes a distinction between pure cases of psychoses of passions (corresponding to delusional disorder) and related cases (occurring in other psychoses, including schizophrenia), we only considered the former cases. We reviewed these clinical cases to examine if delusional features characteristic of schizophrenia (section ‘Delusions characteristic of schizophrenia’) were reported in the clinical cases of delusional disorder (section ‘Clinical cases of delusional disorder’).

## Psychopathology of delusional disorder

The definitions of delusional disorder (paranoia) found in the work of the classical authors all emphasize the presence of a coherent, plausible, chronic delusion, ripe with delusional misinterpretations, with no or few hallucinations, preservation of the personality, and only mood alterations secondary to the delusion (see also Kendler, 2017). Kraepelin (1899, 1903, 1915) and Sérieux and Capgras (1909) describe a general picture of delusional disorder, which they, respectively, call ‘paranoia’ and ‘délire d’interpretation’, as well as a closely related, yet different disorder, i.e. querulomania or ‘délire de revendication’. Kretschmer (2016) and De Clérambault (1942) describe two subtypes of delusional disorder, viz. the ‘sensitive delusion of reference’ and the ‘psychoses of passion’, respectively. Finally, Jaspers (2015) describes two prototypical forms of delusional disorder, one highly systematized and chronic, and another one less systematized and more prone to remission.

Classically, the main symptom of delusional disorder is chronic, systematized, nonbizarre delusions (Jaspers, 2015; Kraepelin, 1903; Sérieux & Capgras, 1909). The delusional beliefs that develop first and organize the subsequent delusional ideas are described as ‘ideo-affective complexes’ (Bleuler, 1912; Sérieux & Capgras, 1909) or ‘ideo-affective nodes’ (De Clérambault, 1942), i.e. ideas intimately associated with strong emotions. Precisely because of their strong emotional dimension, delusional ideas in delusional disorder ‘influence, in their sense, perception, interpretation, memory, fantasy’ (Kraepelin, 1915, p. 1754, our translation). In doing so, they may give rise to delusional misinterpretation, illusions, and false memories. Although these three psychopathological features are not mentioned in current diagnostic manuals as symptoms of delusional disorder, they were described by the classical authors (Jaspers, 2015; Kraepelin, 1903; Sérieux & Capgras, 1909) as the three main symptoms of delusional disorder, in addition to delusion itself. Before discussing each of them in some detail, we will first examine two central characteristics of delusions in delusional disorder, namely their systematization and single-world orientation. These features, though not mentioned as such in DSM-5, are to some extent implied in its conceptualization of delusional disorder, which stresses that ‘functioning is not markedly impaired, and behavior is not obviously bizarre or odd’ and the absence of disorganized speech and behavior (American Psychiatric Association, 2013, pp. 90, 104).

### Systematization and ‘exclusion of the contingent’

Although the level of internal coherence of the delusion varies among patients, what is characteristic across patients with delusional disorder is their clear attempt at systematization (Kraepelin, 1915), that is, the need to organize their delusional ideas in such a way that they are both internally consistent and consistent with other beliefs about the shared social world. Kraepelin (1915) argued that in delusional disorder, ‘contradictions and unclear facts are eliminated as far as possible through a mental work of adjustment, so that the result is a delusional construction which, despite the absurdity and fragility of the foundations, usually does not involve absolute and obvious impossibilities’ (p. 1722, our translation). Concerning the differential diagnosis to schizophrenia (dementia praecox), he argued that in delusional disorder there is an ‘absence of delusion of somatic influence. The idea that external powers can be introduced by telepathy into the mechanism of one’s body, into feelings, into thoughts, into volition, seems to me nothing more than the expression of that volitional disorder […] of the dementia praecox patient. In true paranoia, I have found the delusion of being influenced by poisons in food, but never the idea of being subjected to the direct actions of others. Ideas of harm can be very narrow and implausible, but they are always kept within natural and possible’ (4) (p. 610, our translation). A similar observation was made by Sérieux and Capgras (1909), who argued that delusions ‘normally remain within the realm of the possible, the plausible (malignity, prejudices, thefts, poisonings, etc.); supernatural powers do not intervene’ (pp. 25–26, our translation).

In the most ‘interpretative’ forms of delusional disorder (e.g. those described by Kraepelin [1903] and Sérieux and Capgras [1909]), the level of systematization can be remarkably advanced, leading Rossi Monti (1984) to compare it to the way scientific knowledge is systematized. Drawing on concepts used by Lakatos for scientific theories, Rossi Monti suggested that delusions in delusional disorder can be divided into two parts: (1) delusional beliefs of what he called the ‘protective belt’, i.e. delusional beliefs that the patient to some extent can modify in accordance with empirical data, experience, and objections to ensure the survival of the delusional system and (2) delusional beliefs of the ‘proto-nucleus’, that is, the core, highly affect-laden delusional beliefs that remain unmodifiable during the course of the illness.

The systematization of delusions has been described as an ‘obsession with coherence’, which also involves a characteristic ‘exclusion of the contingent and the fortuitous’ (‘exclusion du contingent et du fortuit’), as Minkowski (1966) put it. ‘The paranoiac’, Nacht and Racamier (1958) argued, ‘has chosen to repress the ineffable […], to eliminate the slightest possibility of it through a tireless perseverance in logic and in the search for causality’ (p. 428, our translation). Thus, patients with delusional disorder exhibit a ‘hyperrational’ approach to information processing, ‘rationally’ ruling out all other possible explanations (Lantéri-Laura et al., 1985).

### Single-world orientation

The patients’ need to harmonize their delusional beliefs with socially acceptable beliefs as well as their incessant search for external confirmation reveal that patients with delusional disorder are heavily occupied with the shared social world. This is a key characteristic of the disorder, common to all varieties of it described by the classical authors. Consistently, Cargnello and Riva, in their review of the German literature on delusional disorder, emphasized the ‘polarization towards reality’ in delusional disorder. This patient, he argued, ‘incessantly sets his delusional theme onto reality; he seeks to find in reality both a justification and an enrichment for his delusion, as well as a reason for the controversy with which he tries to impose it’ (Cargnello & Riva, 1960, p. 161, our translation). Similarly, Sérieux and Capgras (1909) highlighted this dynamic relationship between the patient and reality, as well as their need for social validation of their delusions. We suggest phrasing this key aspect of delusion in delusional disorder as a ‘single-world orientation’, thus differentiating it from the ‘double-world orientation’ characteristic of some delusions in schizophrenia (see section ‘Delusions characteristic of schizophrenia’). Sérieux and Capgras (1909) highlighted something similar concerning the different world orientation in the two disorders, when stating ‘How different [from the schizophrenic] the persecuted-interpreter person is! In him there is no rupture with the external world: he draws from it all the elements of his delusion. Far from being subjected to his insane ideas, he creates them himself, coordinates them; they are an integral part of his ego’ (pp. 282–283, our translation).

### Delusional misinterpretation

Sérieux and Capgras, who term paranoia ‘misinterpretative delusional states’ (délire d’interprétation), describe delusional misinterpretation as a delusional belief ‘which has as its starting point a real sensation [perception] … which, thanks to the associations of ideas linked to character and affectivity, takes on a personal meaning for the patient, through erroneous intuitions and deductions’ (1909, p. 3, our translation). Like delusional perception (see section ‘Delusions characteristic of schizophrenia’), delusional misinterpretation arises from a real perception, which takes on an intense personal significance. Unlike delusional perception, however, delusional misinterpretation is understandable considering the person’s affectivity and thoughts (cf. section ‘Delusions characteristic of schizophrenia’). Moreover, the delusional meaning never emerges as an experience of intrusive and revelatory knowledge, but usually crystalizes gradually over a period of reflection, though with time it ‘becomes more and more intrusive, finally acquiring a certain autonomy’ (p. 220, our translation). As Munro puts it, delusional misinterpretation ‘is an adaptation of a percept to fit in with other delusional beliefs’ (1999, p. 30).

Although delusional misinterpretations are common to a variety of psychoses including schizophrenia, they are, in delusional disorder, multiple and well organized. The most trivial or insignificant events (e.g. a random noise, a chance meeting, a scratch on a suitcase) may, for the patient, acquire the status of unshakable evidence or confirmation of their delusional conviction. Such interpretations may often concern events from the past (Jaspers, 2015; Sérieux & Capgras, 1909) – e.g. Jaspers describes a patient, suffering from a delusion of jealousy, who remembered that he was once told that his wife was ‘a floral arrangement’ (*Blumenarrangement*) and now ‘knows’ that these words were, in fact, referring to her romantic relationship with Mr. Blum (‘Blume’ means flowers in German) (Jaspers, 2015).

### Illusions

Illusions can be described as misinterpretations or misperceptions that, unlike hallucinations, arise from a real object (Jaspers, 1997). In delusional disorder, as evident from the authors’ clinical cases, illusions are determined by the strong affective charge of the delusional conviction – they are, ‘illusions due to affect’, as Jaspers (1997) put it. In delusional disorder, the reality status of illusions is often not questioned by the patients, who instead find in them, as in the case of delusional misinterpretations, only further confirmation of their delusional conviction. For example, Jaspers (2015) reports a case of an illusion, where a patient, after the clinician asked him to write down his curriculum vitae, is convinced that the clinician said ‘periculum vitae’ (i.e. a threat to life) and continued to insist on it. Hallucinations, if present in delusional disorders, play the same confirmatory role (American Psychiatric Association, 2022; World Health Organization, 2024). Correct differentiation between illusion and hallucination is important (Jaspers, 2015; Sérieux & Capgras, 1909) and it is possible that some illusions in delusional disorder are mistaken for hallucinations (Sérieux & Capgras, 1909).

### False memories

False memories are memories retrospectively constructed by the subject. In delusional disorder, such memories concern only events related to the delusional theme, and they can range from a slight alteration of a real memory to fabrication of new memories. Typically, false memories in delusional disorder are reported with an abundance of details (Kraepelin, 1915) and they risk being mistaken for memories of hallucinations (Kraepelin, 1903) – for example, Kraepelin (1903) reports the case of a patient, claiming to be the son of an (imaginary) minister, who remembered his father’s mansion and described it in great detail.

## Delusions characteristic of schizophrenia

Before describing specific types of delusions in schizophrenia, it must be noted that all symptoms, including delusional misinterpretations, illusions, and false memories can be seen in schizophrenia, implying that these symptoms, though described in delusional disorder by the classical authors, are not in themselves decisive in terms of differential diagnosis.

Crucially, the most important symptom shared by delusional disorder and schizophrenia is, of course, delusion. While contemporary research considers delusion as a unitary phenomenon, i.e. a belief meeting certain criteria (i.e. the content of the belief being false or highly unlikely, and the belief being held with subjective certainty and cannot be corrected), researchers, perhaps especially within phenomenological psychopathology, have long argued that certain delusions found in schizophrenia are specific to the disorder, not primarily due to their content but to the way these delusions arise and manifest (Feyaerts et al., 2021).

Jaspers (1997) famously differentiated between *primary delusion* (also called delusion proper), which he considered characteristic for schizophrenia, and *secondary delusions* (also called delusion-like ideas), which can be found in any psychotic disorder, including schizophrenia. Where secondary delusions emerge psychologically understandably from the content of preceding mental states (e.g. affects), primary delusions manifest as immediate and sudden revelations of delusional meaning that cannot be traced back to the content of prior mental states. For Jaspers, understandability of delusion is therefore not really a matter of delusional content but of its mode of emergence – can the content of the delusion be traced back to prior mental states? Those that cannot remain incomprehensible in this specific sense. Delusional perception, i.e. a delusion triggered by a normal perception (e.g. one of Gruhle’s patients reported seeing three marble tables in a café and instantly, upon seeing the tables, knew that the world was about to end [Berze & Gruhle, 1929]), is an example of a primary delusion. According to both Jaspers and Schneider, primary delusion is sometimes preceded by a *delusional mood* (Nielsen et al., 2022), which refers to a state of global or atmospheric insecurity, a diffuse or inexplicable sense that something significant is about to happen, and, increasingly, that whatever is about to happen concerns the patient specifically (Henriksen & Parnas, 2019). Crucially, primary delusions, independently of whether they crystallize on the backdrop of a delusional mood or not, are, for Jaspers, embedded in what he called ‘a transformation in our total awareness of reality’ (1997, p. 95), that is, an altered experiential framework in schizophrenia.

Consistently, Schneider (1959) proposed that certain symptoms had diagnostic specificity for schizophrenia (if no somatic basis could be found), viz. the first-rank symptoms. First-rank symptoms include a set of specific delusions (e.g. delusional perception, thought insertion, withdrawal, or broadcasting, and delusions of control) and hallucinations (i.e. voices discussing the patient or a voice commenting on the patient’s behavior). According to Schneider (1959), delusions of having thoughts inserted or extracted from one’s mind or having one’s will, thoughts, or body steered by an alien force reflect an underlying alteration in the structure of experience, a loss of ego demarcation, which he also referred to as a ‘radical qualitative change’ of consciousness, echoing Jaspers’ remarks about these delusions being embedded in an altered experiential framework in schizophrenia.

More recently, Parnas (2004) proposed to differentiate *autistic-solipsistic* delusions, characteristic of schizophrenia, from *empirical* delusions, which can be seen in various psychotic disorders, including schizophrenia and delusional disorder. Autistic-solipsistic delusion is often colored by metaphysical, eschatological, or charismatic themes, reflecting, again, their embedment in an altered experiential framework (Parnas, 2004). Rather than being epistemic statements about strictly objective matters in the empirical world (i.e. fitting the concept of a belief), autistic-solipsistic delusions concern another realm that looms up before the patient alone. Empirical delusions, by contrast, do not imply an altered experiential framework, and they are strictly concerned with matters pertaining to the shared social world. Unlike autistic-solipsistic delusions, empirical delusions do not violate the laws of causality, noncontradiction, and temporality, and they do not involve extra-physical explanations.

Finally, some patients with schizophrenia have long been argued to exhibit a ‘double-world orientation’ – a phenomenon described already by Bleuler under the heading of ‘double-entry bookkeeping’ (Bleuler, 1950). This phenomenon may explain some of the apparently paradoxical behaviors of patients with schizophrenia in relation to the delusion they entertain (e.g. patients may firmly believe that the food being served by the staff is poisoned but still eat it without any hesitation or resistance). In more recent attempts to define double bookkeeping (Henriksen & Parnas, 2014; Parnas et al., 2021; Stephensen et al., 2023), it has been described as an openness to two different worlds, sometimes existing side-by-side, though, from the perspective of an external observer, these worlds may seem mutually exclusive. One world is the shared social world, and the other is a private-solipsistic and, at times, psychotic world, and patients often seem to experience them as two separate, incommensurable, and nonconflicting realities, allowing them to coexist (Henriksen & Parnas, 2014). Crucially, the ‘other’ world in schizophrenia possesses for the patient a profound significance and an immediate irrefutable reality, which requires no empirical or intersubjective validation. This may explain why many patients with schizophrenia, unlike patients with delusional disorder, do not seek empirical proof or intersubjective validation for their delusions, and also often do not act upon them. Their delusions, quite simply, do not really pertain to something in the shared social world, and others’ perspective on them is often experienced as irrelevant (Parnas et al., 2021).

## Clinical cases of delusional disorder

Several of the original scholars describing delusional disorder (paranoia) also contributed with lengthy clinical cases (De Clérambault, 1942; Jaspers, 2015; Kraepelin, 1906, 1919; Kretschmer, 2016; Sérieux & Capgras, 1909), illustrating important psychopathological characteristics. We reviewed these clinical cases, looking for the presence of delusional features characteristic of schizophrenia. More specifically, we explored if phenomena resembling delusional mood, first-rank symptoms, autistic-solipsistic delusions, and double bookkeeping were reported in these clinical cases. We selected these features of schizophrenia, because, if present in the patients, the symptoms were likely to be mentioned in the cases as all relating to the main symptom of the disorder, namely delusion. Furthermore, authors variously describe these features – or seemingly related ones – as relevant for differentiating delusional disorder from schizophrenia. Notably, there is an overlap between phenomena included in the notion of bizarre delusion and those included in the notions of first-rank symptoms, autistic-solipsistic delusion, and double bookkeeping.

Obviously, we acknowledge the significant limitations involved in reporting psychopathology in written clinical cases. Moreover, the nosological landscape has changed since these cases were originally published. The findings from our reading of these cases must therefore be considered against these limitations. In Table 1, the findings from our review of 46 clinical cases of delusional disorder are presented.

**Table 1.** Occurrence of selected psychopathological features in clinical cases of delusional disorder reported by classical authors. For each author, the number of cases in which these features are present is indicated. These features are examined as psychopathological indicators traditionally associated with schizophrenia and relevant to the experiential context of delusion formation.

	Delusional mood	First-rank symptoms	Autistic-solipsistic delusions	Double bookkeeping
Kraepelin (1906, 1919) 2 cases	Absent	Absent	Absent	Absent
Jaspers (2015) 7 cases	Absent	Absent	Absent	Absent
Kretschmer (2016) 13 cases	5 cases: - case 4* - case 8* - Friedmann’s case 3^+^ - case 10* - case 18	3 cases: - case 4 (thought insertion, thought broadcast, delusion of control)* - Friedmann’s case 3^+^ (delusional perception, thought insertion, thought broadcast, delusion of control) - case 10 (delusional perception)*	1 case: - case 10 (for a limited time)*	1 case: - case 10 (for a limited time)*
Sérieux and Capgras (1909) 20 cases	Absent	3 cases: - case 2 (delusion of control) - case 5 (delusion of control) - case 6 (delusion of control)	Absent	Absent
De Clérambault (1942) 6 cases	Absent	Absent	Absent	Absent

*Note.* Absent = the investigated phenomena were absent in all the cases reported by the author; * = cases described by Kretschmer as occasionally presenting with ‘acute sensitive insanity’, i.e. transient schizophrenia-like manifestations; ^+^ = Kretschmer borrowed this case from Friedmann.

The clear trend is that symptoms traditionally considered characteristic of delusions in schizophrenia are absent in most of the original clinical cases of delusional disorder. Double bookkeeping and autistic-solipsistic delusions were the least present phenomena (both described only in 1 of 46 cases: case 10 in Kretschmer [2016]), followed by delusional mood (described in 5/46 cases: Friedmann’s case 3, and case 4, 8, 10, and 18, all in Kretschmer [2016]), and first-rank symptoms (described in 6/46 cases, always delusional perception or passivity phenomena: Friedmann’s case 3, and case 4, and 10, all in Kretschmer [2016], and cases 2, 5, and 6 in Sérieux and Capgras [1909]). Notably, the very small number of schizophrenia-specific symptoms in the cases of delusional disorder was found primarily in Kretschmer’s (2016) work and can perhaps be explained by his idiosyncratic nosology. Some of his clinical cases of ‘sensitive paranoia’, which we included in the table, also presented with a phase of ‘acute sensitive insanity’, that is transient schizophrenia-like manifestations – these cases account for three cases of delusional mood, two cases of first-rank symptoms, and the single case of autistic-solipsistic delusions and double bookkeeping. In our view, consistent with the view of past and contemporary authors (Ballerini & Rossi Monti, 1990; Del Pistoia, 2008; Priwitzer, 2007), these cases would most likely have been diagnosed within the schizophrenia spectrum today. Overall, delusions characteristic of schizophrenia were described only in a few of the classical clinical cases of delusional disorder.

## Discussion

In this narrative review, we offered a psychopathological characterization of delusional disorder based on descriptions from the scholars, who originally shaped the concept and their relevant clinical cases. Focused on the symptom of delusion, we examined how the scholars originally understood the nature of delusion in delusional disorder and compared this to delusional features considered characteristic of schizophrenia.

Taken together, our findings indicate that delusions in delusional disorder unfold within a largely preserved experiential framework. Across the classical descriptions and clinical cases reviewed, the delusions, however rigid, systematized, and affectively charged, remain embedded in a single shared world, governed by ordinary principles of causality and interpersonal meaning. Alterations of perception, memory, and interpretation are consistently circumscribed to the delusional theme and do not seem to involve any profound transformation of the experiential framework. In this respect, delusional disorder appears to differ qualitatively from schizophrenia in which delusions emerge in the context of more pervasive changes in the experiential framework (Feyaerts et al., 2021).

These psychopathological differences concern not only the manifestation of delusions, but also the experiential framework in which they are embedded, the reality to which they pertain, and the ways in which delusions are enacted. As such, they offer clinically meaningful resources for differential diagnosis that go beyond the mere presence or absence of specific delusional contents.

On this basis, the DSM-5 change, allowing bizarre delusions in delusional disorder, appears problematic. In classical psychopathology, the incomprehensibility of delusion concerned its mode of emergence, implying an altered experiential framework. The mainly content-based definition of bizarre delusion in DSM has principally allowed the possibility for bizarre delusion in psychotic disorders not characterized by an altered experiential framework. Still, the change in DSM-5, allowing bizarre delusion in delusional disorder, lacks empirical and conceptual grounding, contradicts historical accounts of delusional disorders (paranoia), and prior versions of DSM as well as ICD-10 and ICD-11. We suggest it may be worth reconsidering the allowance of bizarre delusion in delusional disorder in the preparation of DSM-6.
